# In vivo monitoring of hepatic glycolipid distribution of *n*-6 ∕ *n*-3 in jugular-vein-cannulated rats as a nutritional research model for monogastric animal

**DOI:** 10.5194/aab-62-437-2019

**Published:** 2019-07-19

**Authors:** Sang-O. Park, Victor A. Zammit

**Affiliations:** 1 College of Animal Life Science, Kangwon National University, Chuncheon, Gangwon-do, 24419 Republic of Korea; 2 Division of Biomedical Sciences, Warwick Medical School, University of Warwick, Coventry, UK

## Abstract

The metabolic distribution via blood from liver of glycerolipids by omega-6 to omega-3 fatty acid (n-6 / n-3) ratio in monogastric animal nutrition is very important. In vivo monitoring technique using jugular-vein-cannulated rats as a nutritional model for monogastric animal can yield important insights into animal nutrition. This study was conducted to determine the effect of different n-6 / n-3 ratios (71:1, 4:1, 15:1, 30:1) on metabolic distribution of glycerolipids newly synthesized and secreted in the liver of the rats and explore the mechanism involved. Regarding ${}^{14}{\mathrm{CO}}_{2}$ released from oxidation of glycerolipid metabolism, it was the highest (22.5 %) in groups with a n-6 / n-3 ratio of 4:1 (P<0.05). The control group showed the highest total glycerolipid level, followed by the 30:1, 15:1, and 4:1 groups in order (P<0.05). When secreted triacylglycerol level of each group was compared with that of the control group, the 4:1, 15:1, and 30:1 groups were decreased by 36.3 %, 20.9 %, and 13.3 %, respectively (P<0.05). Regarding the distribution of phospholipid against total glycerolipid compared to the control group, the 4:1, 15:1, and 30:1 groups were 1.38, 1.29, and 1.17 times higher, respectively (P<0.05). In the comparison of ${}^{14}{\mathrm{CO}}_{2}$ emission against total glycerolipid compared with the control group, the 4:1, 15:1, and 30:1 groups were 1.61, 1.52, and 1.29 times higher, respectively (P<0.05). These results demonstrate that a dietary n-6 / n-3 fatty acid ratio of 4:1 could significantly decrease harmful lipid levels in the blood by controlling the mechanism of metabolic distribution via blood from triglyceride and phospholipid newly synthesized in the liver of cannulated rat.

## 1 Introduction

Humans and other mammals cannot synthesize omega-6 (n-6) or omega-3 (n-3) unsaturated fatty acids because they do not have endogenous enzymes necessary to insert a cis double bond at the n-6 or the n-3 position of a fatty acid. Thus, these essential fatty acids have to be supplied from a diet (Gogus and Smith, 2010; Simopoulos, 2016). The Western diet contains high levels of n-6 but low levels of n-3 fatty acids, with an n-6 to n-3 ratio as high as 20:1. The desirable ratio of n-6 to n-3 is known to be 4:1 or lower (Burghardt et al., 2010; Gómez-Candela et al., 2011; Simopoulos, 2003). The Korean diet contains an n-6 / n-3 ratio of 7.49:1. However, n-6 / n-3 ratio is 71:1 in people who prefer to eat meat because they favor pork belly with high saturated fat (Oh, 2010; Ha and Kim, 2018). Dietary n-6 / n-3 ratio is 15–20:1 in the UK and northern European populations. It is increased to 30:1 in countries where the consumption of edible oil is high, although meat consumption is low due to religious reasons (Oh, 2010). The diet with high n-6 / n-3 ratio can increase the risk of thrombopoiesis and inflammatory response that may lead to atherosclerosis, obesity, and diabetes (Bhardwaj et al., 2016; Gogus and Smith, 2010). Decreasing n-6 / n-3 fatty acid ratio in a diet can reduce the risk of critical cardiovascular diseases (Bucher et al., 2002; Simopoulos, 2008). A previous study has shown that the ratio of n-6 / n-3 fatty acid can influence the growth and body composition of children (Much et al., 2013). Dietary fat could facilitate adaptation to lipid metabolism in the next generation of rats (Chambers et al., 2016; Halfen et al., 2016). Dietary intake with the ratio of n-6 / n-3 dropped to 8:1 or lower from parent generation to the next generation can reduce harmful lipid in the blood, and stimulate growth by activating the metabolism of nutrients (Shin et al., 2017).

Pigs and rats are used as models of nutrition in humans. The gastrointestinal tract of pig is anatomically and physiologically very similar to that of human (Guilloteau et al., 2010). Also, metabolism of nutrients and metabolites absorbed by the digestive tract or metabolism by resident microbiota in pig are close to human (Brugger et al., 2010). However, the in vivo monitoring technique related to this study in pigs is not well known yet. Digestive tract microbiota of pigs, rats, and humans are very similar. Oxidation of short-chain fatty acids can affect microbiotic profile such as *Bifidobactrium* in pigs, rats, and humans (Tomas et al., 2012). The use of the in vivo monitoring method could clearly investigate the mechanism of metabolic distribution via blood from liver of glycerolipid that is newly synthesized and secreted in the liver (Shi and Cheng, 2009; Park, 2016). When investigating the mechanism of lipid metabolism distribution in rats, an in vivo monitoring technique for metabolism of cholesterol secreted from the liver, biosynthesis of triacylglycerol and phospholipid, and metabolism distribution of glycerolipid related to secretion are very important (Rennie et al., 2000; Wang et al., 2010). It is known that as a result of in vivo monitoring of lipid metabolism, fish oil enriched in n-3 can decrease blood lipids due to introduction of acyl moiety of 3-branched-chain fatty acids toward the synthesis of phospholipids as compared to n-6 fatty acid source and pork belly oil (Cao et al., 2004; Shi and Cheng, 2009; Park, 2016). Since post-meal intake of lipoprotein particles and their residues can lead to abundant triglycerides in blood plasma due to secretion of chylomicron in the alimentary canal that is competitive against lipoprotein lipase activity, rats can keep secreting very-low-density lipoprotein triacylglycerol (VLDL-TG), which may aggravate their health (Shi and Cheng, 2009; Wilfling et al., 2013). However, studies on the direct relationship between the ratio of dietary n-6 / n-3 in diet intake and metabolic distribution of hepatic glycerolipids in the monogastric animal are insufficient (Irma et al., 2000; Zhou et al., 2016).

Therefore, the objective of this study was to determine the effect of different n-6 to n-3 fatty acid ratios (71:1, 4:1, 15:1, 30:1) on metabolic distribution of glycerolipids newly synthesized and secreted in the liver of the rats as a nutritional research model to monogastric animal and explore the mechanism involved using an in vivo monitoring technique in rats attached with a jugular-vein cannula.

## 2 Materials and methods

### 2.1 Experimental design

All experimental procedures including animal experiments were in accordance with scientific and ethical regulations provided by the EC Directive of 1986, 86/609/EEC. They were approved by the Institutional Animal Care and Use Committee (IACUC) of Kangwon National University, Republic of Korea (approval no. KNU-16072). Sprague Dawley strain rats (SD, 24 males and 24 females, average weights of 234 g for males and 315 g for females) were purchased from Daehan Bio Link Co. Ltd. Republic of Korea. Animals were kept with a 12 h light–dark cycle at constant temperature (22 ${}^{\circ }$C). They were maintained in individual cages (one animal per cage). They were acclimated to the environment for 1 week before the experiment. They were allowed free access to a chow diet and drinking water. After acclimation for 1 week, rats were randomly assigned to four treatment groups (6 rats per group) for 30 d. Animals were divided into a control group (n-6 / n-3 ratio of 71:1) and three treatment groups (n-6 / n-3 ratio of 4:1, 15:1, and 30:1).

**Table 1:** Fatty acid composition of the used coconut, perilla, corn, and soybean oil (grams per 100 g total fatty acid).

Fatty acid name	Tallow	Corn oil	Perilla oil	Soybean oil
Octanoic acid (C8:0)	–	–	–	–
Decanoic acid (C10:0)	–	–	–	–
Lauric acid (C12:0)	–	0.13	0.04	–
Myristic acid (C14:0)	2.23	0.07	0.02	0.09
Palmitic acid (C16:0)	26.66	12.40	7.84	11.53
Palmitoleic acid (C16:1 n-7)	1.03	0.13	0.27	0.10
Stearic acid (C18:0)	23.23	2.28	3.86	4.84
Oleic acid (C18:1 n-9)	35.27	31.22	14.76	24.36
Linoleic acid (C18:2 n-6)	11.58	51.21	16.88	51.28
Arachidic acid (C20:0)	–	1.03	0.29	0.76
Linolenic acid (C18:3 n-3)	–	1.12	55.67	6.41
Behenic acid (C22:0)	–	0.19	0.38	0.50
Erucic acid (C22:1)	–	–	–	–
Lignoceric acid (C24:0)	–	0.20	–	0.14
Total	100	100	100	100
SFA${}^{1}$	52.12	16.32	12.43	17.85
UFA${}^{2}$	47.88	83.68	87.57	82.15
n-6 / n-3	–	45.68	0.30	8.00
UFA/SFA	0.92	5.13	7.05	4.60

${}^{1}$ SFA: saturated fatty acids. ${}^{2}$ UFA: unsaturated fatty acids. –: not detected.

### 2.2 Experimental diet

This study used an experimental diet mixed with AIN-93 purified diets adjusted to satisfy the level of nutrient requirements for rats. Fatty acid composition of a fat source is indicated in Table 1 while fatty acid composition of the diet is shown in Table 2. Dietary n-6 / n-3 ratio was adjusted to control (71:1 as tallow 70 % plus corn oil 30 %), 4:1 (5.70 % corn oil plus 1.30 % perilla oil), 15:1 (6.70 % corn oil plus 0.30 % perilla oil), or 30:1 (6.20 % corn oil plus 0.80 % soybean oil) for corresponding groups. Diet preparation included processing as pellets and drying until water content was reduced to 10 % in a blower kiln with a temperature of 20 ${}^{\circ }$C. The diets were stored in a low-temperature chamber during experimental periods.

### 2.3 Determination of fatty acid composition

Total fatty acid composition of oils and diets was determined via gas chromatography equipped with a flame ionization detector (GC, Hewlett-Packard 6890). About 50 mg of oils or lipid extracted from diets using chloroform / methanol 2:1 mixture was dissolved in 0.2 mL of hexane containing 5 mg mL${}^{-1}$ of heneicosanoate (C21:0) as the internal standard. In the next stage, sample was saponified using 1 mL of methanolic 0.5 N NaOH solution and methylated by boiling for 15 min with 1 mL of BF${}_{3}$ methanol. After cooling at room temperature, 3 mL of hexane was added and the phase was determined by GC. Fatty acid methyl ester was separated using an RTX-2330 capillary column (60 m × 0.25 mm × 0.2 µm, Restek, USA). Oven temperature was set 60 ${}^{\circ }$C (5 min). Temperature was increased at a rate of 6 ${}^{\circ }$C min${}^{-1}$ up to 240 ${}^{\circ }$C (5 min). Helium (1 mL min${}^{-1}$) was the carrier gas. Injector temperature and detector temperature were 230 and 250 ${}^{\circ }$C, respectively. Identification of individual fatty acid methyl ester was performed on the basis of retention time of PUFA No. 2 animal source (Supelco, Bellefonte, PA, USA) (Kieliszek et al., 2018).

### 2.4 In vivo monitoring

To conduct the in vivo monitoring experiment, this study selected six rats per treatment group of the next-generation rats after completion of the feeding experiment and after a total of 24 rats were randomly allotted four treatment groups. After installing a jugular cannula, the in vivo monitoring technique was used to determine the distribution of lipid metabolism as described previously (Park, 2016). Sprague Dawley male rats each weighing 460 g were purchased from Daehan Bio Link Co. Ltd. To accelerate secretion of VLDL-TG newly synthesized in the liver, 10 % fructose solution was ingested by each rat as drinking water 48 h before blood collection. After killing these lipoprotein donor animals, 10 mL of blood was collected from each animal through abdominal arteries. Blood plasma was then prepared and used for lipoprotein isotope labeling as described previously (Wang et al., 2010; Umpleby, 2015). An in vivo cannulation technique was utilized in accordance with the European laboratory animal handling license (SCT-W94058) acquired by Park (2016). A mixture of ketamine (ketamine hydrochloride, 50 mg, Yuhan Chemical Inc., South Korea) and Rompun (Rompun, xylazine hydrochloride, 23.32 mg, Bayer Korea, South Korea) at a ratio of 3:1 was injected into white rats (0.15 mL per 100 g white rat). After administering anesthetic by intraperitoneal injection, 0.4 mL of antibiotics (solution of 0.6 g of amoxicillin dissolved in 2 mL of citric saline) was administered by intraperitoneal injection. Polyethylene cannula (ID 0.63 mm, OD 1.19 mm; Silastic tubing, VWR–Dow Corning no. 508-003, Midland, MI, USA) sterilized by exposing to the right-side jugular vein was injected into each rat. After spraying acrylamide (Dales Pharmaceuticals) over the surgical field of each rat and stitching its surgical wound, the head of the rat was moved toward its cannula using a catheter syringe before completely stitching the cut. In order to determine blood flow, 0.3 mL citric saline was injected into the cannula of each rat 3 times per day. Rats were then given a 5 d recovery period (Thrivikraman et al., 2002; Park, 2016). After adding heparin to very-low-density lipoprotein (VLDL remnants) of the blood obtained from donor animal in order to accelerate its breakdown, VLDL remnants were cultured alongside a reaction reagent at 37 ${}^{\circ }$C for 30 min. First, plasma was obtained after centrifuging the cultured fluid at 5000 g for 15 min. To maintain a constant density (d=1017 g mL${}^{-1}$) of the blood from which the separation of VLDL remnants was possible, NaCl/KBr (d=1346 g mL${}^{-1}$) solution in the same amount as the blood was slowly injected along a test tube wall. For the separation of VLDL remnants, a Sorvall centrifuge (as high as 29 000 g, RC 6 Plus, Thermo Fisher Scientific Inc., UK), a vacuum high-speed centrifuge separator that could keep the temperature at 12 ${}^{\circ }$C, was used for 20 h (Iwasaki et al., 2005; Nakajima et al., 2011; Wilfling et al., 2013; Park, 2016). Following a 20 h centrifugation, approximately 2 mL of VLDL-remnants-containing material was separated before passing through a size-exclusion chromatograph (Sephadex G25) to remove KBr (potassium bromide) from the solution. Fractions were then collected. Again, VLDL remnants were separated from these fractions to dispense lipopolysaccharide (LPS). From each quick-fit small tube, [${}^{3}$H] cholesteryl oleoyl ether of 9 µL and cholesteryl [${}^{14}$C] oleate of 7.5 µL were taken out and dried under nitrogen gas. After adding approximately 1 mL acetone, the solution was very slowly mixed. Radiochemicals, [${}^{3}$H] and [${}^{14}$C], were purchased from Amersham International (Amersham, Bucks., UK). CETP (cholesterol ester transfer protein) of 1.5 mL was poured into the abovementioned test tube and mixed with foams for 15 min using nitrogen gas. After adding reaction reagents to the dispensed 3.6 mL LPS before making a mixture of CETP isotope, the mixture was put into a test tube mixed with foams under nitrogen gas for 15 min. The mixture was incubated in a heating system (DB-3, technique, UK) at 37 ${}^{\circ }$C for 3 h to label lipoprotein. After finishing the incubation, NaCl/KBr solution was again injected slowly along the upper wall of the test tube. Repeating the aforementioned process, VLDL remnants were separated and the LPS dispensed via the final separation was filtered through a 0.45 µm filter immediately before use (Iwasaki et al., 2005; Nakajima et al., 2011; Wilfling et al., 2013; Umpleby, 2015; Park, 2016). The abovementioned manufactured VLDL remnants labeled with isotope were measured using a scintillation counter (Packard 1600TR, Hewlett Packard, Palo Alto, CA, USA). The amount to be injected into each animal was adjusted to [${}^{14}$C] 300 000 dpm and [${}^{3}$H] 300 000 dpm. After VLDL remnants were injected via a 60 min jugular cannula, rats were placed into a desiccator chamber (pump: Masterflex model 7524-50; Cole-Parmer Instrument Co. Ltd. breath-sampling bag: Laboratory for Expiration Biochemistry Nourishment Metabolism Co., Ltd.) in which the air (respiratory rate of 5 L min${}^{-1}$) was supplied in order to conduct a respiratory metabolism test. During in vivo monitoring, rats were fixed in the desiccator chamber for respiratory metabolism test (fatty acid oxidation) (Lang et al., 2001; Wilfling et al., 2013). At 15 min after injecting VLDL remnants, Triton WR 1339 solution of 1.0 mL in saline solution was poured using a cannula. Hydrolysis of VLDL-TG secreted from the circulation was minimized. After completion of a respiratory metabolism test, pentobarbitone (50 mg kg${}^{-1}$) was intraperitoneally injected into anesthetized rats. Liver and blood samples from these anesthetized animals were collected and lipid was extracted to measure ${}^{14}$C-labeling in livers, secreted TGs, and phospholipid. Carbon dioxide emitted from the process was used to keep a constant flow (10 L min${}^{-1}$) of air via the desiccator chamber. It was captured in a 100 mL mixture of ethanolamine and ethylene glycol monomethyl ether (1:2, v/v) (Iwasaki et al., 2005; Wilfling et al., 2013; Mattis et al., 2015).

**Table 2:** Fatty acid composition of the experimental diets (grams per 100 g total fatty acid).

Fatty acid name	Dietary n-6 / n-3 ratio${}^{1}$			
	Control	4:1	15:1	30:1
Octanoic acid (C8:0)	10.12	–	–	–
Decanoic acid (C10:0)	6.61	–	–	–
Lauric acid (C12:0)	26.25	–	–	–
Myristic acid (C14:0)	19.92	–	–	–
Palmitic acid (C16:0)	8.54	11.68	12.63	12.84
Palmitoleic acid (C16:1 n-9)	–	–	–	–
Stearic acid (C18:0)	3.66	2.60	2.40	2.70
Oleic acid (C18:1 n-9)	3.22	28.23	30.91	31.07
Linoleic acid (C18:2 n-6)	21.39	46.09	50.69	51.70
Arachidic acid (C20:0)	–	–	–	–
Linolenic acid (C18:3 n-3)	0.30	11.40	3.36	1.69
Behenic acid (C22:0)	–	–	–	–
Erucic acid (C22:1)	–	–	–	–
Lignoceric acid (C24:0)	–	–	–	–
Total	100	100	100	100
SFA${}^{2}$	75.10	14.28	15.03	15.54
UFA${}^{3}$	24.90	85.72	84.97	84.46
n-6 / n-3	71.30	4.04	15.08	30.59
UFA/SFA	0.05	6.00	5.65	5.43

${}^{1}$ Control, purified diet containing 7.0 % coconut oil; 4:1, 5.70 % corn oil plus 1.30 % perilla oil; 15:1, 6.70 % corn oil plus 0.30 % perilla oil; 30:1, 6.20 % corn oil plus 0.80 % soybean oil. ${}^{2}$ SFA: saturated fatty acids, ${}^{3}$ UFA: unsaturated fatty acids. –: not detected.

### 2.5 Sampling and thin-layer chromatography analysis

This study anesthetized rats with the abovementioned method. A constant body temperature was maintained for these animals using infrared rays. By keeping a constant body temperature, the abdominal cavity of the rat was dissected and spread before promptly taking 3 mL of blood from the rat's artery. The lobe of each rat's left-side liver was taken out via the cold-clamping technique using a stainless-steel triangle-shaped clamp. The rest of the liver, hind-limb muscles, and fat tissues of rats were collected after going through cold clamping under liquid nitrogen. They were stored until analysis. Lipids were taken from tissues and lipid fractions were separated via thin-layer chromatography (TLC, Merck KGaA, Darmstadt, Germany). In this study, repeated experiments were conducted on numerous occasions by the nature of the research because each experimental rat had to have a jugular cannula via surgery and was forced to take a certain period of recovery. For that reason, tests could not be implemented on a lot of animals at the same time (Park, 2016; Cid-Hernández et al., 2018).

### 2.6 Statistical analysis

All statistics analyses of this study used the SAS program. For the purpose of statistical analysis, average and standard deviations were obtained for each treatment group and then analysis of variance was conducted. Significance was evaluated using Duncan's multiple range test. The repeatability of the obtained test results indicated a probability of 95 % (P<0.05).

**Table 3:** ${}^{14}{\mathrm{CO}}_{2}$ emission and incorporation of [${}^{3}$H] and [${}^{14}$C] into liver and plasma at 60 min after injection of LPS (d<1.019) labeled with cholesteryl [${}^{14}$C] oleate in jugular-vein-cannulated rats.

	Incorporation of [${}^{3}$H] and [${}^{14}$C] label (% of injected dose)				
n-6 / n-3	Plasma		Liver		${}^{14}{\mathrm{CO}}_{2}$
	[${}^{3}$H]	[${}^{14}$C]	[${}^{3}$H]	[${}^{14}$C]	emission
Control (71:1)	1.16±0.02	$28.15\pm 0.55^{\text{1a}}$	92.25±2.33	$35.77\pm 0.92^{\text{a}}$	$8.11\pm 0.16^{\text{d}}$
4:1	1.20±0.02	$15.14\pm 0.33^{\text{d}}$	92.74±2.18	$25.36\pm 0.38^{\text{d}}$	$22.54\pm 0.45^{\text{a}}$
15:1	1.15±0.01	$18.05\pm 0.35^{\text{c}}$	92.46±2.77	$29.08\pm 0.79^{\text{c}}$	$19.77\pm 0.38^{\text{b}}$
30:1	1.18±0.02	$26.22\pm 0.52^{\text{b}}$	92.21±3.01	$33.01\pm 0.76^{\text{b}}$	$15.53\pm 0.25^{\text{c}}$

${}^{1}$ Mean ± standard error. ${}^{\text{a,b,c,d}}$ Values within the same column with different superscripts are significantly different (n=6, P<0.05).

## 3 Results

### 3.1 Glycerolipid metabolism significantly high in an animal fed a diet containing lower ratio of n-6 / n-3

The incorporation rate of ${}^{14}$C-labeling lipid (CLL) and ${}^{14}{\mathrm{CO}}_{2}$ emission rate into glycerolipid with regards to the distribution of total lipids in the blood and livers of the rats are shown in Table 3. The incorporation rate of ${}^{3}$H-labeling lipid (HLL) in the liver of each rat was higher than that of the CLL in the liver and blood. The incorporation rate of the HLL in the liver was high (at 92.21 %–92.74 %) while the incorporation rate in the blood was low (at 1.15 %–1.20 %). The incorporation rate into the HLL in the liver or blood did not show significant differences among treatment groups. For CLL in livers of rats, the incorporation rate was high in the following order: control group >30:1 group >15:1 group >4:1 group. The incorporation rate in the liver ranged from 25.36 % to 35.77 %, which was significantly higher (P<0.05) than that in the blood (15.14 %–28.15 %). Regarding the ${}^{14}{\mathrm{CO}}_{2}$ emission rate by glycerolipid metabolism of rats, the 4:1 group had the highest rate while the control group had the lowest rate. Differences in ${}^{14}{\mathrm{CO}}_{2}$ emission rate among treatment groups were significant (P<0.05). The ${}^{14}{\mathrm{CO}}_{2}$ emission rate of rats ranged from 8.11 % to 22.54 %. This study found that animals fed with diets having different ratios of n-6 / n-3, especially those fed with a diet having a n-6 / n-3 ratio of 4:1, showed faster glycerolipid metabolism.

**Table 4:** ${}^{14}{\mathrm{CO}}_{2}$ emission and tissue accumulation of [${}^{14}$C] lipid in jugular-vein-cannulated rats.

			Tissue [${}^{14}$C] lipid accumulation		
			(% of absorbed dose g${}^{-1}$ of tissue)		
n-6 / n-3	Absorption of	${}^{14}{\mathrm{CO}}_{2}\mathrm{emission}$			
	[${}^{14}$C] limit (%)	(% of absorbed	Adipose	Liver	Muscle
		dose)	tissue		(hind leg)
Control (71:1)	$66.52\pm 1.77^{\text{1a}}$	$23.08\pm 0.51^{\text{d}}$	$1.80\pm 0.02^{\text{a}}$	$1.35\pm 0.01^{\text{a}}$	$0.80\pm 0.01^{\text{a}}$
4:1	$55.75\pm 1.39^{\text{d}}$	$33.81\pm 0.98^{\text{a}}$	$0.43\pm 0.01^{\text{d}}$	$0.28\pm 0.01^{\text{d}}$	$0.17\pm 0.002^{\text{d}}$
15:1	$59.12\pm 1.71^{\text{c}}$	$30.01\pm 0.84^{\text{b}}$	$0.90\pm 0.01^{\text{c}}$	$0.72\pm 0.01^{\text{c}}$	$0.40\pm 0.01^{\text{c}}$
30:1	$63.02\pm 1.89^{\text{b}}$	$27.11\pm 0.62^{\text{c}}$	$1.33\pm 0.01^{\text{b}}$	$1.03\pm 0.01^{\text{b}}$	$0.68\pm 0.01^{\text{b}}$

${}^{1}$ Mean ± standard error. ${}^{\text{a,b,c,d}}$ Values within the same line with different superscripts are significantly different (n=6, P<0.05).

### 3.2 ${}^{14}$CO${}_{2}$ emission rate and ${}^{14}$C-labeling lipid accumulation rate in tissues of the rats

After installing jugular cannula, the ${}^{14}{\mathrm{CO}}_{2}$ emission rate and the ${}^{14}$C-labeling lipid accumulation rate in tissues of the rats in experiment diet groups were different to each other. Results are shown in Table 4. The absorption rate of CLL and the ${}^{14}{\mathrm{CO}}_{2}$ emission rate showed significant differences among treatment groups (P<0.05). The absorption rate of CLL in the control group was significantly higher than that in any other group. The absorption rate of CLL in the liver was significantly lower in the order of 4:1 group <15:1 group <30:1 group (P<0.05). In the meantime, ${}^{14}{\mathrm{CO}}_{2}$ emission rate of the 4:1 group was the highest. The ${}^{14}{\mathrm{CO}}_{2}$ emission rate in the 4:1, 15:1, or 30:1 group was 1.45 to 1.17 times higher than that in the control group. In the adipose tissue of the control group, the rate of CLL storage was 1.13 to 4.18 times higher than that in the 4:1, 15:1, or 30:1 groups, supporting results of CLL the absorption rate.

**Table 5:** Distribution of glycerolipid in livers of jugular-vein-cannulated rats.

		% Secreted			
	Total glycerolipids	Phospholipid	TG	Phospholipid/total	${}^{14}{\mathrm{CO}}_{2}$/[${}^{14}$C] total
n-6 / n-3	(% of cholesterol	(% of total	(% of total	glycerolipid (%)	glycerolipid (%)
	[${}^{14}$C]-oleate	glycerolipid	triacylglycerol		
	metabolized in liver)	labeled)	labeled)		
Control (71:1)	$81.88\pm 2.46^{\text{1a}}$	$14.80\pm 0.38^{\text{d}}$	$57.81\pm 1.68^{\text{a}}$	$30.87\pm 0.07^{\text{d}}$	$24.72\pm 0.63^{\text{d}}$
4:1	$73.57\pm 1.85^{\text{c}}$	$37.04\pm 1.07^{\text{a}}$	$33.21\pm 0.82^{\text{d}}$	$43.18\pm 0.83^{\text{a}}$	$42.74\pm 0.95^{\text{a}}$
15:1	$73.69\pm 1.92^{\text{c}}$	$30.01\pm 0.67^{\text{b}}$	$44.77\pm 0.90^{\text{c}}$	$40.05\pm 0.88^{\text{b}}$	$38.17\pm 1.14^{\text{b}}$
30:1	$76.54\pm 1.69^{\text{b}}$	$19.73\pm 0.38^{\text{c}}$	$52.55\pm 1.35^{\text{b}}$	$36.03\pm 0.68^{\text{c}}$	$32.56\pm 0.83^{\text{c}}$

${}^{1}$ Mean ± standard error. ${}^{\text{a,b,c,d}}$ Values within the same line with different superscripts are significantly different (n=6, P<0.05).

### 3.3 Lower ratio of n-6 / n-3 accelerate oxidation quotient of fatty acid

After installing jugular cannula, cholesteryl ester rates metabolized in the liver collected from the product of blood and liver esterification of the rats fed with diets having different n-6 / n-3 ratios were different from each other. Results are shown in Table 5. Values presented in Table 4 are percentages (%) of the metabolism volume based on ${}^{3}$H recovery rate to compensate different measured values caused by lipid metabolism. With regard to gross glycerolipids (cholesteryl [${}^{14}$C] oleate metabolized from the liver, %), the control group had the highest level, followed by the 30:1, 15:1, and 4:1 groups in order (P<0.05). There was no significant difference in this level between the 4:1 and 15:1 groups. Regarding phospholipid secretion rate, 4:1, 15:1, and 30:1 groups were 1.33 to 2.50 times higher than the control group (P<0.05). Regarding triglyceride secretion rate, the 4:1, 15:1, and 30:1 groups were significantly reduced (P<0.05) by 42.55 %, 22.56 %, and 9.10 %, respectively, compared to the control group. For the distribution rate of phospholipid against total glycerolipid, the 4:1, 15:1, and 30:1 groups were 1.40, 1.29, and 1.17 times higher (P<0.05), respectively, compared to the control group. Regarding ${}^{14}{\mathrm{CO}}_{2}$ emission rate against ${}^{14}$C-labeled total glycerolipid, the 4:1, 15:1 and 30:1 groups were 1.73, 1.54, and 1.32 times higher (P<0.05), respectively, than the control group. These results demonstrate that dietary intake of n-6 / n-3 at a ratio of 4:1 can especially accelerate the oxidation quotient of fatty acid in the rats.

**Figure 1:**
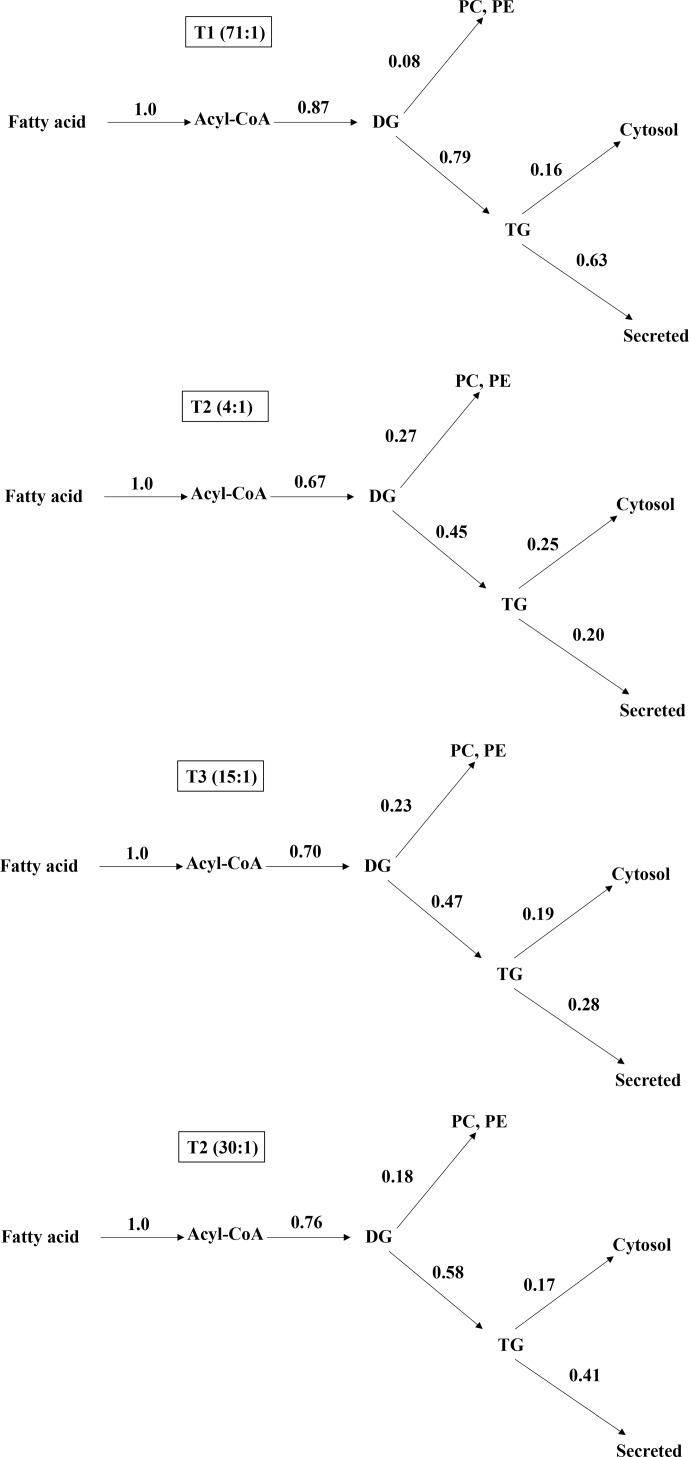
Distribution of acyl moiety fluxes for oxidation and glycerolipid synthesis in the liver of jugular-vein-cannulated rats (n=6). DG, diacylglycerol; TG, triglyceride; PC, phosphatidylcholine; PE, phosphatidylethanolamine.

### 3.4 Lower ratio of n-6 / n-3 increase metabolic distribution of phospholipid

After installing jugular cannula, contributions at metabolic branch points regarding lipogenesis in the rats fed with experimental diets containing different n-6 / n-3 ratios were different in each other. Results are shown in Fig. 1. Regarding the ${}^{14}$C-labeling TG secretion rates from the liver, the control, 1.2:1, 8:1, and 30:1 groups were 72.41 %, 29.85 %, 40.00 %, and 53.95 %, respectively. As the rate of n-6 / n-3 was lowered from 30:1 to 4:1, the distribution of the TG was decreased. For distribution rates of phospholipid, the control, 4:1, 15:1, and 30:1 groups were 9.20, 40.30, 32.86, and 23.68 %, respectively. Thus, when the rate of n-6 / n-3 is decreased from 30:1 to 4:1 the distribution of phospholipid is increased.

## 4 Discussion

The reason why research on the distribution mechanism of fatty acid in the liver uses ${}^{3}$H and ${}^{14}$C at the same time is as follows. [${}^{3}$H]-cholesteryl oleoyl ether was used to compensate the metabolism rate of VLDL-TG that was synthesized and secreted from lipid metabolism. It was generated from the liver after injecting ${}^{14}$C-labeled material. In such a case, when lipid metabolism in the body progresses, the liver incorporation rate of ${}^{3}$H-labeled material shows a much higher value than that of ${}^{14}$C-labeled material. However, the ${}^{3}$H incorporation rate into the blood is so much lower or almost zero (Wilfling et al., 2013; Grevengoed et al., 2014; Park, 2016; Kollareth et al., 2018). Unlike cholesteryl [${}^{14}$C] oleate, [${}^{3}$H]-cholesteryl oleoyl ether does not go through a metabolism path like the liver. Thus, more than 90 % of it will be left in the liver. With the use of these two isotope labeling substances, it is possible to measure the mechanism of distribution of glycerolipid in the liver more accurately (Shi and Cheng, 2009; Park, 2016). Cholesteryl [${}^{14}$C] oleate is metabolized into [${}^{14}$C] oleic acid and cholesterol in the liver. In the end, the oleic acid will go through an oxidation and esterification process before being used for the synthesis of new VLDL-TG. The newly synthesized VLDL is moved into the blood in 30 min. By using cholesteryl [${}^{14}$C] oleate, it is possible to measure the amount of newly synthesized VLDL-TGs secretion in the blood (Coleman and Lee, 2004; Chen et al., 2008; Umpleby, 2015). In the literature of in vivo monitoring of the metabolism of hepatic fatty acid, the dynamics of emission of ${}^{14}{\mathrm{CO}}_{2}$ show a difference gradually. This is due to direct production of ${}^{14}{\mathrm{CO}}_{2}$ in the liver and oxidation of ${}^{14}$C ketone body in epithelial tissues (Huynh et al., 2014; Park, 2016). Theoretically, ${}^{14}$C ketone body could be combined into ${}^{14}$C-TG in lipogenesis tissues. However, its amount is reported to be less than 2 % 1 h after injecting a ${}^{14}$C labeling substance. In a in vivo monitoring study, the amount of ${}^{14}{\mathrm{CO}}_{2}$ emission is much more important than the oxidation of ${}^{14}$C ketone body (Lang et al., 2001; Villanueva et al., 2009; Hodson and Frayn, 2011; Umpleby, 2015). The present study found that the control group had the highest incorporation rate of glycerolipid into the liver and blood from the newly injected CLL while animals in the group with diets containing a n-6 / n-3 ratio of 4:1 had the lowest rate. However, results for the ${}^{14}{\mathrm{CO}}_{2}$ emission rate were the opposite. These results demonstrate that glycerolipid metabolism is activated when the ratio of n-6 / n-3 in the diet of animals is maintained at 4:1. It is reported that n-3 from fish oil regulates hepatic glycerolipid metabolism by increased peroxisomal fatty acid oxidation of rats compared to n-6 fatty acids, which supports this finding (Constantin et al., 2013; Grevengoed et al., 2014).

It is reported that the rate of emission of ${}^{14}{\mathrm{CO}}_{2}$ is increased linearly for the first hour after feeding the diet to fasting rats and the rate is significantly lower in the normal diet intake group. The rate of absorption of CLL for 5 h since then was increased in the indefinitely feeding group of rats. Thus, in order to compensate such differences and suggest more exact data, it would be desirable to indicate the rate of emission of ${}^{14}{\mathrm{CO}}_{2}$ and the rate of CLL storage in percent (%) against absorption volume as shown in Table 5 (Villanueva et al., 2009; Hodson and Frayn, 2011; Constantin et al., 2013; Huynh et al., 2014). The reason why lower n-6 / n-3 ratio leads to lower accumulation rate of CLL in tissues and higher rate of ${}^{14}{\mathrm{CO}}_{2}$ emission might be associated with the higher storage of biological lipids in the control group. This indicates that the newly injected CLL is used for lipid storage in fatty tissues rather than oxidation (liver, muscles, and brown fat) (Zammit et al., 1999; Wang et al., 2010; Grevengoed et al., 2014). Muscles are known to play a critical role in removing circulative triglyceride for their oxidation (Chen et al., 2008; Grevengoed et al., 2014; Umpleby, 2015). Results of the present study showed that the control group of rats had the highest rate of CLL accumulation in hind-leg muscles, 1.18 to 4.70 times higher than those in the 4:1, 15:1, and 30:1 groups. These results suggest that the consumption of a diet containing a n-6 / n-3 ratio of 4:1 in animals can accelerate oxidation of lipids and decrease lipid storage in biological tissues.

In general, when the rate of esterification of fatty acid by the liver is lowered, most phospholipids will stay in the liver and maintain the structure of liver cell membrane properly while saving energy for phospholipid synthesis (Irma et al., 2000; Hall et al., 2012; Huynh et al., 2014). The rate of distribution of glycerolipid into the blood from the liver from newly injected ${}^{14}$C-labeling substance, in the case of triglyceride, was low in the treatment group with different n-6 / n-3 ratio. However, the rate of secretion of phospholipid and the rate of emission of ${}^{14}{\mathrm{CO}}_{2}$ were the opposite. Findings of this study reveal that, when the n-6 / n-3 ratio is getting lowered to 8:1 or lower, secretion of triglyceride is decreased in the distribution of the newly synthesized glycerolipid. At the same time, secretion of phospholipid is increased and emission of ${}^{14}{\mathrm{CO}}_{2}$ is increased, thus lowering hazardous lipids in the blood.

Compared with the control group, when the n-6 / n-3 ratio was lowed, harmful lipids were decreased. This would have multiple reduction effects on the direction of distribution of the acyl moiety that can lead to the path of distribution of acyl-CoA, diacylglycerol, phospholipid, and TG (Cao et al., 2004; Coleman and Lee, 2004; Koves et al., 2009; Wilfling et al., 2013; Grevengoed et al., 2014). Therefore, an increase in triglyceride being stored in cells caused by increased secretion of acyl-CoA towards oxidation in general can make the same contribution. The synthesis of glycerolipid is the main route of fatty acid metabolism in the rat's liver and their tissues, although rats might also make use of newly synthesized fatty acid and seek esterification of some glycerols (Zammit et al., 1999; Shi and Cheng, 2009; Wang et al., 2010; Grevengoed et al., 2014). Since the distribution of fatty acids in the liver between formation of acylcarnitine for fat oxidation and synthesis of esterification defines the rate of oxidation of the same fatty acids, the distribution of liver fatty acids between oxidation and esterification is very important (Umpleby, 2015). Metabolism of fatty acid in rats that had the diet for animals progresses in the direction of synthesis of triglyceride and phospholipid. Triglyceride-containing very-low-density lipoprotein, which is synthesized in the liver and is secreted into the blood, is used as a substrate for lipoprotein lipase, a breakdown enzyme of lipoprotein in adipose tissue of animals (Koves et al., 2009; Hodson and Frayn, 2011; Ebihara et al., 2013; Grevengoed et al., 2014).

## 5 Conclusions

We performed in vivo monitoring of hepatic glycolipid distributions from dietary n-6 / n-3 by using a rat with jugular-vein cannula as a nutritional model of monogastric animal. In this study, the diet group with lower n-6 / n-3 ratio of 4:1 resulted in the lowest secretion of triacylglycerol levels but the highest secretion of phospholipid levels and ${}^{14}{\mathrm{CO}}_{2}$ emission in glycerolipid distribution via blood from liver of rats. Optimal n-6 / n-3 fatty acid ratio of 4:1 can be used to prepare novel diets that can directly improve human and animal health.

## Data Availability

The data sets are available upon request from the corresponding author.
